# The Antioxidant and Anti-Inflammatory Properties of *Anacardium occidentale* L. Cashew Nuts in a Mouse Model of Colitis

**DOI:** 10.3390/nu12030834

**Published:** 2020-03-20

**Authors:** Rosalba Siracusa, Roberta Fusco, Alesso Filippo Peritore, Marika Cordaro, Ramona D’Amico, Tiziana Genovese, Enrico Gugliandolo, Rosalia Crupi, Antonella Smeriglio, Giuseppina Mandalari, Salvatore Cuzzocrea, Rosanna Di Paola, Daniela Impellizzeri

**Affiliations:** 1 Department of Chemical, Biological, Pharmaceutical and Environmental Sciences, University of Messina, 98166 Messina, Italy; rsiracusa@unime.it (R.S.); rfusco@unime.it (R.F.); aperitore@unime.it (A.F.P.); rdamico@unime.it (R.D.); tgenovese@unime.it (T.G.); egugliandolo@unime.it (E.G.); asmeriglio@unime.it (A.S.); gmandalari@unime.it (G.M.); dimpellizzeri@unime.it (D.I.); 2 Department of Biomedical, Dental and Morphological and Functional Imaging University of Messina, Via Consolare Valeria, 98125 Messina, Italy; cordarom@unime.it; 3 Department of Veterinary Sciences, University of Messina, 98168 Messina, Italy; rcrupi@unime.it; 4 Department of Pharmacological and Physiological Science, Saint Louis University School of Medicine, Saint Louis, MO 63104, USA

**Keywords:** colitis, *Anacardium occidentale* L., cashew nuts, inflammation, oxidative stress, cytokines

## Abstract

Background: *Anacardium occidentale* L. is a tropical plant used for the treatment of inflammatory diseases. The goal of the present work was to investigate the anti-inflammatory and anti-oxidant potential of oral administration of cashew nuts (from *Anacardium occidentale* L.) in a mouse model of colitis. Methods: Induction of colitis was performed by intrarectally injection of dinitrobenzene sulfonic acid (DNBS). Cashew nuts were administered daily orally (100 mg/kg) in DNBS-injected mice. Results: Four days after DNBS, histological and macroscopic colon alterations as well as marked clinical signs and increased cytokine production were observed. Neutrophil infiltration, measured by myeloperoxidase (MPO) positive immunostaining, was correlated with up-regulation of adhesion molecules ICAM-1 and P-selectin in colons. Oxidative stress was detected with increased malondialdehyde (MDA) levels, nitrotyrosine, and poly ADP-ribose polymerase (PARP) positive staining in inflamed colons. Oral treatment with cashew nuts reduced histological, macroscopic damage, neutrophil infiltration, pro-inflammatory cytokines and MDA levels, as well as nitrotyrosine, PARP and ICAM-1, and P-selectin expressions. Colon inflammation could be related to nuclear factor (NF)-kB pathway activation and reduced manganese superoxide dismutase (MnSOD) antioxidant activity. Cashew nuts administration inhibited NF-kB and increased MnSOD antioxidant expressions. Conclusions: The results suggested that oral assumption of cashew nuts may be beneficial for the management of colitis.

## 1. Introduction

An imbalance of immune response CD4^+^ Th1 against type 2 Th2 in favor of Th1 cells seems to be a decisive pathogenic mechanism in chronic inflammatory bowel disorders (IBDs) such as Crohn’s disease (CD) and ulcerative colitis (UC) [1]. This theory is supported by studies on IBD patients, where an increased proinflammatory cytokines, chemokines, and adhesion molecules expression was observed in mucosal biopsies [2].

In recent years, numerous studies focused on reactive oxygen and nitrogen species (ROS, RNS) as etiologic elements for IBD [3]. The intestinal area is a main place for origination of pro-oxidants, whose formation is principally due to the existence of an excess of microbes, food constituents, and communications between immune cells [4]. Likewise, the anti-oxidant capability of IBD patients is diminished, even in the asymptomatic stage of the illness [5]. To scavenge RNS, intestinal cells require some enzymatic and non-enzymatic antioxidants, such as superoxide dismutase (SOD), but disproportionate production of RNS augments lipid peroxidation (LP) and could lessen antioxidant protections [6]. It should be noticed that oxidative stress (OS) associated to immune activation and inflammation could contribute to fibrosis and tissue injury that distinguish bowel diseases [7].

Existing therapies for IBD include corticosteroids, sulfasalazine, immunosuppressive agents, and biological drugs including anti-TNF-α (alpha tumor necrosis factor) antibodies [8]. However, the adverse effects linked to these medications after persistent treatment periods and the excessive relapse rate limit their usage [9]. A great fraction of patients with IBD show no clinical improvement with the current cures [10]. Recent works have proposed that antioxidants administration from diverse sources, with further anti-inflammatory action may be valuable in the treatment of IBD since inflammation with OS contribute to tissue damage [11,12].

The increased use of medicinal plants to treat medical conditions was associated to the higher demand of pharmacological studies in order to broaden scientific knowledge and better describe the mechanisms related to the plant functionality. Various clinical and preclinical reports have reported on the antioxidant and antimicrobial properties of plant composites and their byproducts [13]. 

*Anacardium occidentale* L. (family *Anacardiaceae*), normally notorious as the cashew tree, is a widely distributed local Brazilian species. Currently, it is registered in the National Program of Medicinal Plants and Herbal Medicine of Italy’s unique health system for therapeutic purposes [13]. Different portions of *A. occidentale* (stem, leaves, fruits, and flowers), have elucidated diverse ethnopharmacological applications. It is common in popular medicine to treat diabetes, infections, as well as hemorrhage and diarrhea. Silva et al. [14] proved a wide antimicrobial activity of the ethanolic extract of flowers of *A. occidentale*, and this effect was related to the presence of alkaloids, saponins, phenolic acids, and tannins.

Daily balanced intake of nuts is important for health since they contain proteins and beneficial fatty acids together with essential nutrients [15]. Consumption of polyphenol-rich food, fresh fruit, and vegetables could neutralize the oxidative effect of ROS [16]. Several studies have reported cholesterol- lessening effect [17], as well as cardio-protective properties of almonds [18] and reduction of inflammatory mediators associated to the ingestion of nuts [19].

Cashew nut is one of the four well-known nuts in the world known for its elevated nutritional importance and inimitable taste. As part of a healthy balanced diet, cashew nut can modulate the risk of cardiovascular diseases, including stroke and metabolic syndrome [20]. A previous study evaluated the effects of diet together with industrial by-products such as from cashew (*Anacardium occidentale* L.) fruit on the intestinal healthiness and lipid metabolism of rats with diet-induced dyslipidaemia [21]. The ingestion of cashew nut also improved the outcome of dyslipidemic rats [22] as well as oral treatment with the ethanolic extract of flowers regulated systemic inflammatory response during a model of cecal ligation and puncture (CLP) of diabetic mice [23]. However, there are few studies which investigate the biological actions of cashews in non-healthy people.

Based on these reports, the goal of the present work was to investigate the anti-inflammatory and anti-oxidant potential of oral administration of cashew nuts in a mouse model of colon inflammation induced by intrarectally injection of dinitrobenzene sulfonic acid (DNBS). This model supports human Crohn’s disease-like features, including nuclear factor κB (NF-κB)-dependent Th1 activation [24].

## 2. Materials and Methods

### 2.1. Materials

Cashew kernel samples (*Anacardium occidentale* L.) obtained from West Africa were used in the study. All chemicals were taken from Sigma-Aldrich and stock solutions prepared in saline (0.9% NaCl; Baxter, Milan, Italy). Solvents were purchased from Merck (Darmstadt, Germany).

### 2.2. Characterization of Cashew Samples

#### 2.2.1. Moisture Determination

The moisture content of cashew samples (10 g) was estimated according to the Association of Official Analytical Chemists (AOAC) Official Method 925.40 (1995) [25]. The results of moisture content are expressed as percentage of fresh weight.

#### 2.2.2. Total Protein Determination

Total nitrogen in cashew samples (0.1 g) was determined by micro-Kjeldahl according to the AOAC method 950.48 (1995) [25]. Protein content was calculated as N× 6.25 and expressed as a percentage fresh weight. 

#### 2.2.3. Lipid Content Determination

Lipid extraction of cashew samples (10 g) was performed using a Soxhlet apparatus according to the AOAC Official Method 948.22 (1995) [25]. The results are expressed as percentage of fresh weight.

#### 2.2.4. Dietary Fiber Determination

Dietary fiber content of cashews (1 g) was determined according to the AOAC Official Method 985.29 (1997) [26]. Results are expressed as percentage of fresh weight.

#### 2.2.5. Total Soluble Sugars

The soluble sugar content of cashew samples was detected according to the method developed by Dubois et al. (1956) and modified by Agrawal et al. (2015) [27,28].

#### 2.2.6. Ash Determination

Ash determination of cashew samples (5 g) was carried out according to the AOAC Official Method 923.03 (1995) [25]. Ash content was expressed as percentage of fresh weight.

#### 2.2.7. Polyphenols Extraction

A cashew extract was prepared as described by [29] with some modifications. Briefly, 10 g of cashews were extracted three times with 20 mL of *n*-hexane (Merck, Darmstadt, Germany) for 6 h under agitation in order to discard the fat. After filtration, the residue was mixed with 100 mL of methanol/HCl (Merck, Darmstadt, Germany) 0.1% (v/v), sonicated for 15 min, and centrifuged (5000× *g*, 10 min, 4 °C). The extraction was repeated twice more, followed by concentration of the methanol fraction in a rotary evaporator. The residue was then dissolved in MilliQ water (40 mL) and extracted four times with 40 mL of ethyl acetate (Merck, Darmstadt, Germany). After combining the organic phases, Na_2_SO_4_ was used for 20 min to dry them.

#### 2.2.8. Total Phenols (TP) Determination

The total phenolic content was determined according to the Folin-Ciocalteu method described by [29] with some modifications. Briefly, 50 µL of sample was added to Folin-Ciocalteu reagent (500 µL) followed by deionized water (450 µL). After 3 min, 500 µL, 10% w/v of sodium carbonate (Sigma-Aldrich, Milan, Italy) was added and samples were left in the dark at room temperature for 1 h, vortexing every 10 min. Absorbance was measured at 785 nm using a UV-Vis spectrophotometer (Shimadzu UV-1601, Kyoto, Japan). Results are expressed as mg of gallic acid equivalents (GAE)/100 g of fresh weight.

### 2.3. Animals

Sprague Dawley male rats (250 gr, Envigo, Milan, Italy) and male CD1 mice (25 g; Envigo, Milan, Italy) were housed in a well-ordered locality (room 22 ± 1 °C 12-h dark/light cycles) with ordinary rodent chow and water. The animals adjusted to these circumstances in one week. Messina University Review Board for the animal care endorsed the research. All animal experiments agree with the new Italian regulations (D.Lgs 2014/26), EU regulations (EU Directive 2010/63).

### 2.4. Carrageenan (CAR)-Induced Paw Edema (Preliminary Data)

CAR - induced paw edema was performed as previously indicated by a subplantar injection of CAR (0.1 mL/rat of a 1% suspension in saline) (Sigma-Aldrich, Milan, Italy) into the right hind paw [30]. Increase in paw volume (mL) was measured using a plethysmometer (Ugo Basile, Varese, Italy) immediately prior to the CAR injection and at 30 min and each hour for 6 h.

### 2.5. Preparation of Cashew Samples

Cashew kernel samples from West Africa were ground and then dissolved in saline prior to oral administration.

### 2.6. Colitis Induction

Colitis was triggered in mice by an intrarectally injection of DNBS (4 mg in 100 μL of 50% ethanol per mouse) (Sigma-Aldrich, Milan, Italy) as previously indicated and the mice were sacrificed on day 4 post-induction [31].

### 2.7. Experimental Groups

In the first step, we studied the acute anti-inflammatory effects of cashew nuts and a possible dose-response in a classical acute model of inflammation. Rats were divided into different groups:

CAR + vehicle: rats were subjected to CAR-induced paw edema, as described above and administered vehicle (saline);

CAR *+* cashew nuts (30mg/kg): same as the CAR+vehicle group but cashew nuts at dose of 30 mg/kg was administered instead of vehicle orally 30 min before CAR injection;

CAR *+* cashew nuts (60mg/kg): same as the CAR+vehicle group but cashew nuts at dose of 60 mg/kg was administered instead of vehicle orally 30 min before CAR injection;

CAR *+* cashew nuts (100mg/kg): same as the CAR+vehicle group but cashew nuts at dose of 100 mg/kg was administered instead of vehicle orally 30 min before CAR injection;

Sham operated groups received saline instead of CAR and were treated orally with saline or nuts.

Doses were given on the basis of a dose-response study. We started with 30 mg/kg dose based on a previous work on nuts [32].

The administration of cashew nuts was done orally for 4 days based on our previous studies on the effects of antioxidants on mouse model of colitis induced by intrarectal DNBS injection [24,33,34].

After a preliminary data on dose response, we decided to carry on with the experiments by evaluating the chronic effect of cashew nuts at a dose of 100 mg/kg (higher dose which showed a significant reduction of paw volume increase) in an experimental model of colitis by instillation of DNBS in the colon. In particular, mice were divided into the following groups:

DNBS +saline: mice were subjected to DNBS administration described as above, and saline was administered orally every 24 h, for 4 days, starting from 1 h after the administration of DNBS.

DNBS+cashew nuts: mice were subjected to DNBS administration described as above, and cashew nuts (100 mg/kg) was administered orally every 24 h, for 4 days, starting from 1 h after the administration of DNBS.

Sham operated groups: vehicle solution (saline) or cashew nuts were orally administered for 4 days.

Since no significant change was found between sham groups, we present data of sham+vehicle groups only.

### 2.8. Evaluation of Colon Damage

After collection, the whole colon was delicately laved with saline, untied by a longitudinal incision, and microscopically examined. Macroscopic damage grade was scored by two autonomous observers [35]. During sacrifice, the colon length (starting above the anus to the top of the cecum), was measured.

### 2.9. Histological Examination

For histological evaluation, tissues were treated with hematoxylin and eosin staining and the damage was scored semi-quantitatively from 0 to 4 as previously described [24] in a blinded fashion, by two qualified pathologists using a Leica DM6 microscope (Leica Microsystems SpA, Milan, Italy) associated with Leica LAS X Navigator software (Leica Microsystems SpA, Milan, Italy).

### 2.10. Malondialdehyde (MDA) Assay

MDA levels were quantified in colon tissue 4 days after DNBS administration as previously reported [31] to detect lipid peroxidation.

### 2.11. Immunohistochemical Localization of Cell Adhesion Molecules (ICAM-1, P-Selectin), Poly (ADP-Ribose Polymerase) (PARP), Nitrotyrosine and Myeloperoxidase (MPO)

Immunohistochemical analysis was performed as previously described at 4 days after DNBS administration [31]. The sections were incubated overnight with primary antibodies: anti-ICAM-1 mouse polyclonal antibody (1:100 in PBS, v/v, Santa Cruz Biotechnology SCB, D.B.A, Milan, Italy), anti-P-selectin mouse polyclonal antibody (1:100 in PBS, v/v, SCB, D.B.A, Milan, Italy), anti-PARP mouse polyclonal antibody (1:100 in PBS, v/v, SCB, D.B.A, Milan, Italy), anti-nitrotyrosine rabbit polyclonal antibody (1:200 in PBS, v/v, Millipore, D.B.A, Milan, Italy), anti-MPO (Neomarkers, 1:200 D.B.A, Milan, Italy). All sections were washed with PBS and then treated as previously reported [31].

Five stained sections from each mouse were scored in a blinded fashion and observed using a Leica DM6 microscope (Leica Microsystems SpA, Milan, Italy) following a typical procedure [36]. The histogram profile is related to the positive pixel intensity value obtained [37].

### 2.12. Western Blots for IKB-α, NF-κB p65, Inducible Nitric Oxide Synthetase (iNOS) and Manganese Superoxide Dismutase (MnSOD)

Cytosolic and nuclear extracts were prepared as previously described on colon tissues [38]. The following primary antibodies were used: anti-IκB-α (SCB, 1:500 #sc1643, D.B.A, Milan, Italy), anti-NF-κB p65 (SCB; 1:500 #sc8008), anti-iNOS (BD Transduction Laboratories, 1:500), anti-MnSOD (Millipore, 1:500, Cat 06-984, D.B.A, Milan, Italy) in 1× phosphate-buffer saline (Biogenerica srl, Catania, Italy), 5% w/v non-fat dried milk, 0.1% Tween-20 at 4 °C overnight. Membranes were incubated with peroxidase-conjugated bovine anti-mouse IgG secondary antibody or peroxidase-conjugated goat anti-rabbit IgG (Jackson ImmunoResearch, West Grove, PA, USA; 1:2,000) for 1 h at room temperature. Anti-β-actin or anti-lamin A/C (D.B.A, Milan, Italy) antibodies were used as controls. Protein expression was analyzed as previously reported [38].

### 2.13. Cytokines Measurements

TNF-α and interleukin (IL)-1β levels were evaluated in the colon tissues collected at 4 days after DNBS by using a colorimetric commercial kit (R&D Systems, Milan, Italy).

### 2.14. Statistical Evaluation

All values are stated as mean ± standard error of the mean (SEM) of N observations. N represents the number of animals. For histology and immunohistochemistry, the photographs are the outcomes of at least three independent experimentations. A *p*-value of less than 0.05 was significant. One- or two-way ANOVA, followed by a Bonferroni post-hoc test for multiple comparisons were used.

## 3. Results

### 3.1. Composition of Cashew Kernel Samples

Table 1 reports the nutritional profile of cashew kernel samples from West Africa. In agreement with the chemical composition of other tree nuts, cashew kernels contain a high proportion of lipid (44.70%), followed by carbohydrate (36.66%) expressed as sum of soluble sugars and fiber and protein (21.01%). Interestingly, the total phenolic content, expressed as mg/100 g of sample, was 69.64.

### 3.2. Acute Effects of Cashew Nuts on Car Induced Paw Edema: Preliminary Data

**Figure 1:**
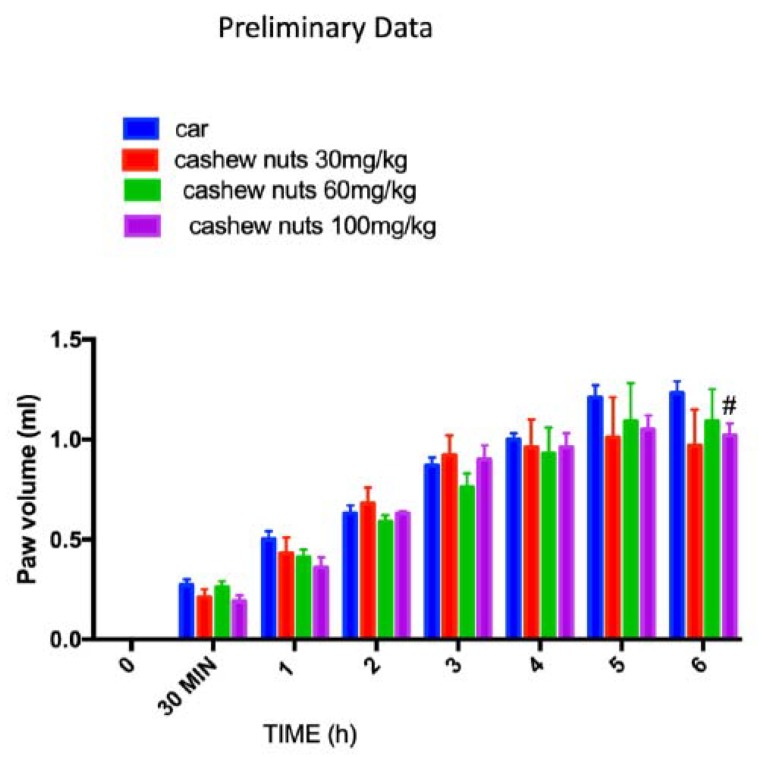
Preliminary data of acute effects of cashew nuts at different doses on car-induced paw edema. The animals were treated at different doses respectively 30, 60, and 100 mg/kg. Values = means ± standard error of the mean (SEM) of six animals in each group; ^#^ *p* < 0.05 vs. Carrageenan (CAR).

To investigate which dose of cashew nuts could have an anti-inflammatory activity, we first studied the acute effects of cashew nuts on a classical model of inflammation such as car-induced paw edema. We tested three different doses 30, 60, and 100 mg/kg. Intraplantar car injection caused an important time-dependent increase in paw volume in CAR-injected rats until 6 h. Cashew nuts at doses of 30 and 60 mg/kg were not able to reduce, in a significant way, the paw edema, whereas the higher dose of 100mg/kg was able to reduce, even if statistically significant only at 6 h post CAR, the paw inflammation (Figure 1).

### 3.3. Chronic Effects of Cashew Nuts on Colitis Degree

**Figure 2:**
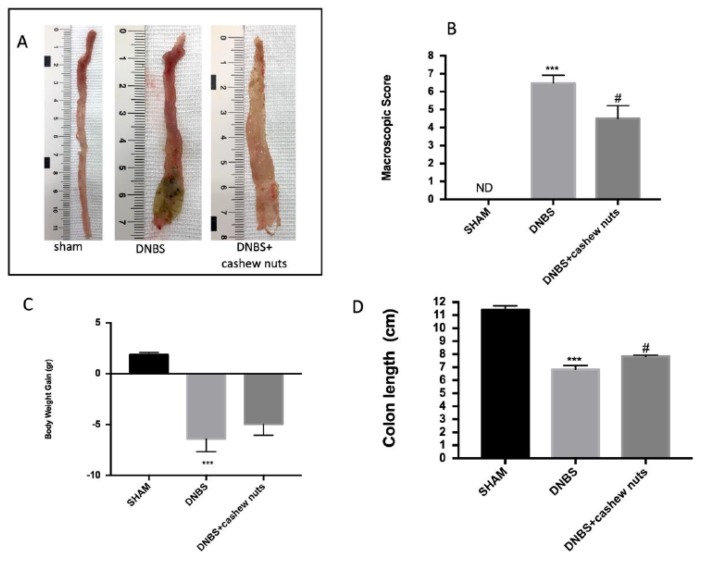
The effects of cashew nuts on macroscopic damage, body weight increase, and colon length after dinitrobenzene sulfonic acid (DNBS) injection. Macroscopic damage in sham, DNBS, DNBS + cashew nuts groups (**A** and **B**). Body weight increase in all groups (**C**). Colon length (**D**). Values = means ± SEM of six animals in each group; *** *p* < 0.001 vs. sham; ^#^ *p* < 0.05 vs. DNBS.

In the colons from shams, no macroscopic change was observed. Four days after intrarectal administration of DNBS, the colon was soft, with mucosal congestion, liquid stool, and ulcerations (Figure 2A,B). The oral treatment of cashew nuts at 100 mg/kg ameliorated inflammation degree in DNBS mice compared to sham (Figure 2A,B). In addition, all mice had a diminution in body weight and colon length compared to sham groups. The oral treatment with cashew nuts was able to ameliorate clinical signs of colon inflammation (Figure 2B–D).

### 3.4. The Chronic Effects of Cashew Nuts on Histological Colon Damage

**Figure 3:**
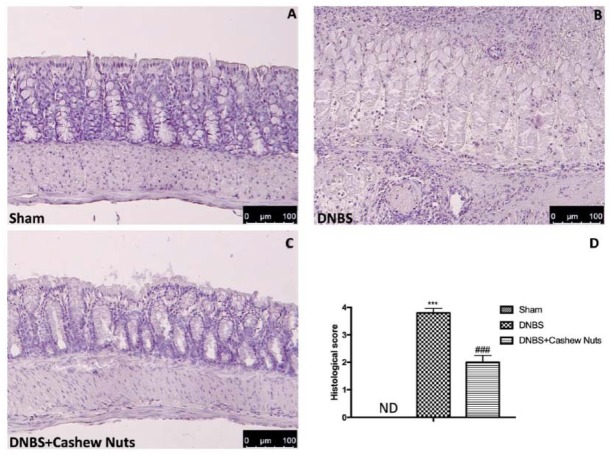
The effects of cashew nuts on histological damage after DNBS injection. Histological analysis was evaluated in sham (**A**), DNBS (**B**), DNBS+cashew nuts (**C**). Histological score was measured (**D**). Images are figurative of at least three independent experiments. Values = means ± SEM of six animals in each group; *** *p* < 0.001 vs. sham; ^###^ *p* < 0.001 vs. DNBS.

No histological modification was observed in the colon tissue from sham mice (Figure 3A,D). A clear leukocyte infiltration, necrosis, and edema was observed in the sections of colon from DNBS-injected mice (Figure 3B,D). The oral treatment with cashew nuts reduced histological impairments (Figure 3C,D).

### 3.5. The Chronic Effects of Cashew Nuts on Neutrophil Infiltration, Cytokines Levels and Lipid Peroxidation

**Figure 4:**
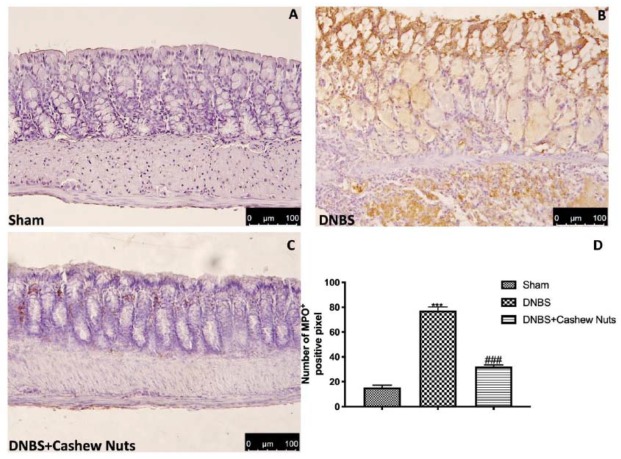
The effects of cashew nuts on myeloperoxidase (MPO) expression after DNBS injection. Immunohistochemistry for MPO was evaluated in sham (**A**), DNBS (**B**), DNBS+cashew nuts (**C**). The results are expressed as % of positive pixels (**D**). Images are figurative of at least three independent experiments. Values = means ± SEM of six animals in each group; *** *p* < 0.001 vs. sham; ^###^ *p* < 0.001 vs. DNBS.

**Figure 5:**
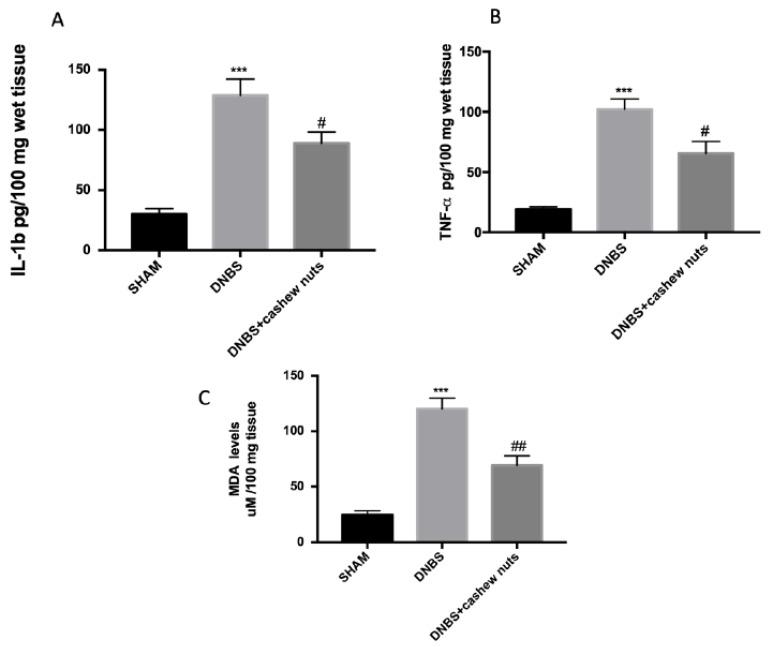
The effects of cashew nuts on cytokine and MDA levels. IL-1β (**A**), TNF-α (**B**), MDA (**C**) levels were examined in all groups. Cashew nuts treatment reduces cytokines and MDA levels. Values = means ± SEM of six animals in each group; *** *p* < 0.001 vs. sham; ^#^ *p* < 0.05 vs. DNBS. ^##^ *p* < 0.01 vs. DNBS.

The colon injury was also illustrated by an increase in MPO positive staining, an indicator of the neutrophil amassing in the colon. In addition, colons from DNBS mice showed increased proinflammatory cytokines levels and lipid peroxidation provoked by neutrophil derived superoxide anion measured by MDA assay. In that regard, mice subjected to DNBS showed increased MPO positive staining (Figure 4B,D), increase of TNF-α, IL-1β (Figure 5A,B), and MDA levels (Figure 5C) compared to sham groups (Figure 4A,D and Figure 5A–C). The oral treatment with cashew nuts at 100 mg/kg significantly reduced positive staining for MPO (Figure 4C,D), cytokines levels such as TNF-α and IL-1β (Figure 5A,B), and caused a reduction of MDA (Figure 5C).

### 3.6. The Chronic Effects of Cashew Nuts on Nitrotyrosine and PARP Expression

**Figure 6:**
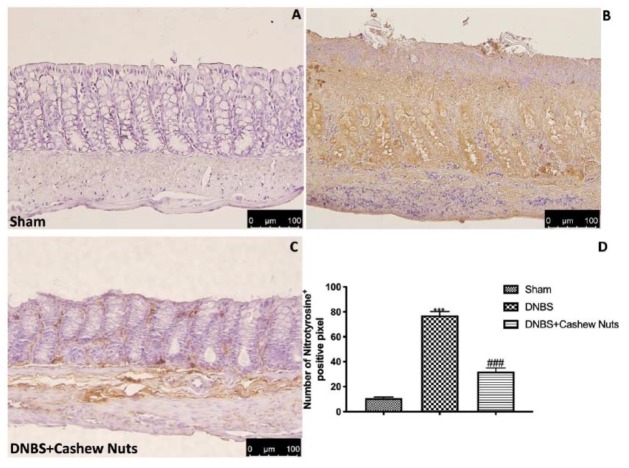
The effects of cashew nuts on nitrotyrosine expression after DNBS injection. Immunohistochemistry for nitrotyrosine was evaluated in sham (**A**), DNBS (**B**), DNBS+cashew nuts (**C**). Results are expressed as % of positive pixels (**D**). Images are figurative of at least three independent experiments. Values = means ± SEM of six animals in each group; *** *p* < 0.001 vs. sham; ^###^ *p* < 0.001 vs. DNBS.

**Figure 7:**
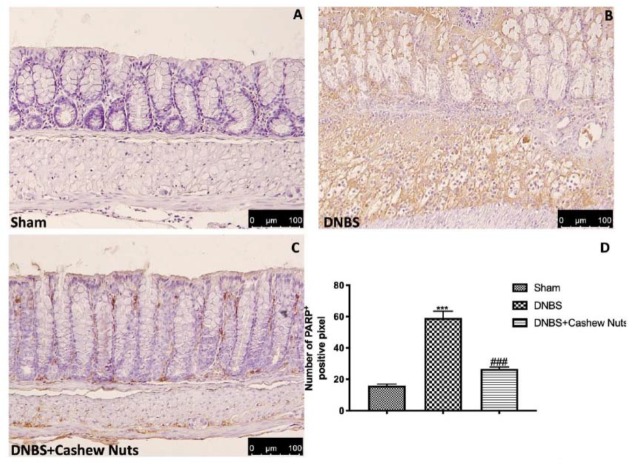
The effects of cashew nuts on PARP expression after DNBS injection. Immunohistochemistry for PARP was evaluated in sham (**A**), DNBS (**B**), DNBS+cashew nuts (**C**). The results are expressed as % of positive pixels (**D**). Images are figurative of at least three independent experiments. Values = means ± SEM of six animals in each group; *** *p* < 0.001 vs. sham; ^###^ *p* < 0.001 vs. DNBS.

Colon sections from sham mice (Figure 6A,D) did not mark for nitrotyrosine, unlike sections from DNBS-injected mice showing a robust positive staining for nitrotyrosine (Figure 6B,D). In addition, increased PARP positive staining was observed in the colon tissues from DNBS-injected mice compared to sham (Figure 7A,B,D). The oral treatment with cashew nuts at 100 mg/kg significantly reduced positive staining for nitrotyrosine and PARP (Figure 6 and Figure 7C,D).

### 3.7. The Chronic Effects of Cashew Nuts on ICAM-1 and P-Selectin Expression

**Figure 8:**
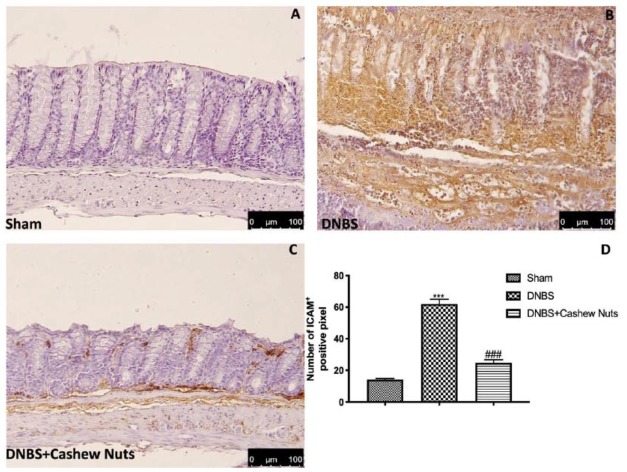
The effects of cashew nuts on ICAM-1 expression after DNBS injection. Immunohistochemistry for ICAM-1 was evaluated in sham (**A**), DNBS (**B**), DNBS+cashew nuts (**C**). The results are expressed as % of positive pixels (**D**). Images are figurative of at least three independent experiments. Values = means ± SEM of six animals in each group; *** *p* < 0.001 vs. sham; ^###^ *p* < 0.001 vs. DNBS.

**Figure 9:**
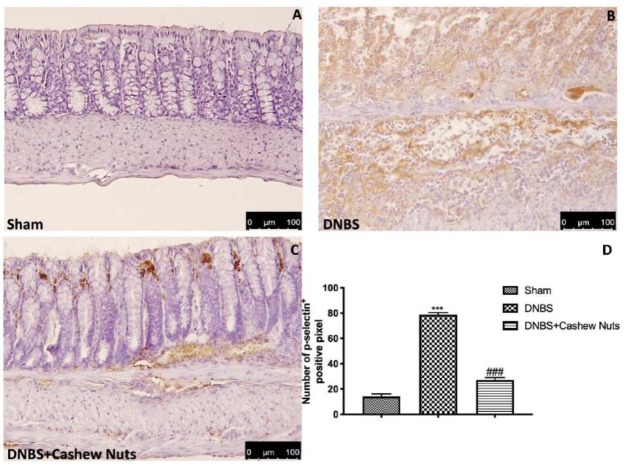
The effects of cashew nuts on P-selectin expression after DNBS injection. Immunohistochemistry for P-selectin was evaluated in sham (**A**), DNBS (**B**), DNBS+cashew nuts (**C**). The results are expressed as % of positive pixels (**D**). Images are figurative of at least three independent experiments. Values = means ± SEM of six animals in each group; *** *p* < 0.001 vs. sham; ^###^ *p* < 0.001 vs. DNBS.

Intestinal expression of ICAM-1 and P-selectin are also implicated during cell enrollment. No positive staining was found in sham mice (Figure 8 and Figure 9A,D). Positive staining for ICAM-1 (Figure 8B,D) and for P-selectin (Figure 9B,D) were prominently increased in tissues from DNBS-injected mice. The oral treatment with cashew nuts was able to reduce, in a significant way, the positive staining for ICAM-1 and P-selectin (Figure 8 and Figure 9C,D).

### 3.8. The Chronic Effects of Cashew Nuts on NF-κB, IKB-α, iNOS and MnSOD

**Figure 10:**
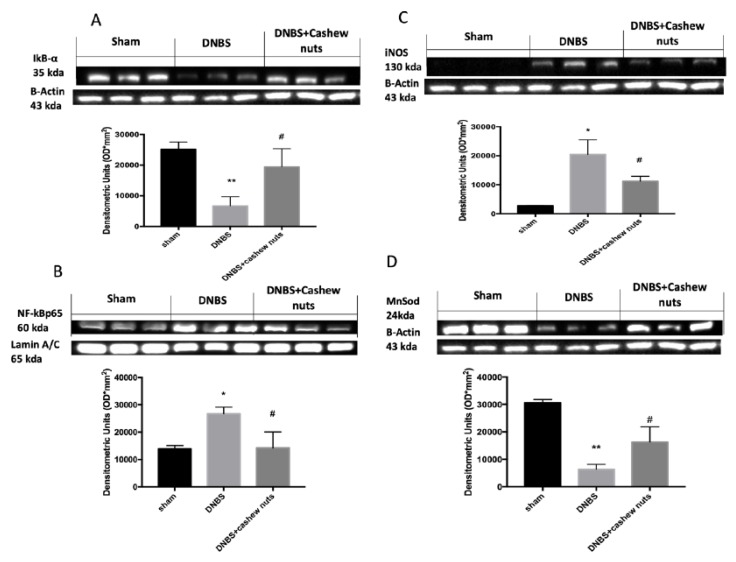
Western blots for NF-kB, IKB-α, iNOS, and MnSOD. Representative Western blots for IKB-α degradation (**A**), NF-κB translocation (**B**), iNOS (**C**), and MnSOD expression (**D**) were performed. A demonstrative blot of lysates (six animals/group), with a densitometric analysis for all animals is showed. The results in A, B C, D = means ± SEM of six animals in each group. * *p* < 0.05 vs. sham; ** *p* < 0.01 vs. sham ^#^ *p* < 0.05 vs. DNBS.

Western blots for NF-κB pathway were performed to further investigate which signaling pathway connected to ROS could be implicated in the beneficial effects of cashew nuts. NF-κB p65 levels increased 4 days after DNBS injection compared to sham groups (Figure 10B). Moreover, in colon samples from sham, a normal expression of IκB-α was identified, whereas IκB-α levels were extensively reduced in colon tissues of DNBS mice (Figure 10A). The treatment with cashew nuts was able to reduce nuclear translocation of NF-kB p65 as well as IKB-α degradation (Figure 10A,B). In addition, to better investigate the antioxidant effects of cashew nuts we also evaluated the expression of iNOS a proinflammatory enzyme related to NF-kB activation and responsible to nitric oxide (NO) production and the expression of an antioxidant enzyme MnSOD (Figure 10C,D). DNBS injection caused increased expression of iNOS as well as a reduction of antioxidant defense with diminished MnSOD expression (Figure 10C,D). The oral treatment with cashew nuts was able to reduce iNOS as well as increase MnSOD expression (Figure 10C,D).

## 4. Discussion

In this study we demonstrated for the first time the antioxidant and anti-inflammatory effects of cashew nuts in an experimental model of colitis. The results showed that cashew nuts had preventive effects on DNBS-induced intestinal inflammation in mice, illustrating its potential as an efficient food for the management of human IBD. In particular, we have shown that cashew nuts attenuated DNBS-induced colitis in mice as evinced by decreases in macroscopic damage, inflammatory infiltration and oxidative stress, as well as improvements in the colon cytoarchitecture.

Various data have proposed that NF-kB is a principal protagonist in the control of many genes with inflammation during experimental colitis [24]. Activation of NF-κB can be inducted by stimuli such as lipopolysaccharides, pro-inflammatory cytokines, and DNA damaging agents [39].

IBD is characterized by an increase in IL-1β, IL-6, IL-17,TNF-α, prostaglandins, and NO, which may damage barrier function and muscle contraction [40]. According to preceding reports, the release of proinflammatory cytokines IL-1, IL-6, and TNF-α is regulated by intracellular signal transduction such as the NF-κB pathway. Once triggered, NF-kB also controls survival and cell proliferation as well as adhesion molecules (i.e., ICAM) and growth factors expressions, which impact the development of intestinal inflammation. According to our results, we can hypothesize that cashew nuts exercises intestinal anti-inflammatory activity due to the inhibition of the NF-kB signaling pathway.

During intestinal inflammatory events, cells and macrophages can produce enormous quantities of TNF-α. This can intensify the expression of adhesion molecules such as VCAM-1 and ICAM-1 in endothelial cells, so meaningfully stimulating the infiltration of leukocytes into the intestinal mucosa. In this study we demonstrated that cashew nuts caused a reduction on NF-kB pathway, pro-inflammatory TNF-α, and IL-1β levels as well as a decreased expression of ICAM-1 and P-selectin that may be a result of the enhancement in intestinal injury. The helpful effects of cashew nuts were also verified in the histopathological analysis of the colonic sections and in the reduction of MPO positive staining, an important marker of neutrophil infiltration.

These results are in agreement with a previous study in which the oral treatment with pistachios reduced myocardial tissue injury, adhesion molecules such as ICAM-1, P-selectin expression, neutrophil infiltration, proinflammatory cytokines as TNF-α and IL-1β production, nitrotyrosine and PAR formation, NF-κB expression, and apoptosis [41]. Mandalari et al. also examined the protective effects of natural almond skin powder in mice subjected to experimental colitis [34].

It has been recognized that chronic intestinal inflammation is associated with oxidative and nitrosative stress [42], which are involved in several human diseases, including IBD. Extensive evidences suggest that IBD is linked with a discrepancy between ROS and antioxidant activity that creates oxidative stress as the consequence of either ROS over-production or a decreased antioxidant activity. High levels of ROS have damaging effects that can affect lipids, proteins, and nucleic acids by instigating fragmentation products that can cause lipid peroxides development, enzymatic alteration, and DNA strand break products [43].

In addition, iNOS is involved in the pathogenesis of bowel inflammation and increased volumes of NO released by iNOS could react with superoxide to form peroxynitrite, thus provoking injurious alterations in protein structure and functionality [44]. Based on these findings, in this study we demonstrated that oral treatment with cashew nuts was able to significantly reduce lipid peroxidation by MDA levels, nitrotyrosine production, and PARP activation, INOS as well as increased antioxidant MnSOD expression. Previous studies also showed that anacardic acids from cashew nuts reduced oxidative stress by diminishing MDA concentration and increasing the levels of reduced glutathione (GSH) and catalase (CAT) enzymatic activity [45] as well as the treatment with cashew apple juice (from *Anacardium occidentale* L.) augmented antioxidants level inhibiting the inflammatory response exerted by ROS production in mouse models of wound excision and xylene-induced ear edema [46].

## 5. Conclusions

In conclusion, in an experimental model of colitis, cashew nuts were able to alleviate the clinical signs, histological damage, neutrophil infiltration, oxidative stress, the secretion of pro-inflammatory cytokines such as IL-1β, TNF-α, as well as to reduce iNOS, ICAM-1, and P-selectin expressions likely through the inhibition of ROS induced-NF-kB activation and increased antioxidant capacity. Thus, the administration of cashew nuts could have beneficial action for the treatment of IBD.

## Figures and Tables

**Table 1:** Nutritional profile of cashew kernel samples from West Africa. Results are expressed for 100 g of cashew kernel samples.

Nutrients	Units	Cashew Kernel
Moisture	g	4.86
Protein	g	21.01
Lipids (total)	g	44.70
Dietary fibre (total)	g	3.86
Sugars (total)	g	32.80
Ash	g	2.68
Total phenols	mg	69.64

## Abbreviations

DNBS	dinitrobenzene sulfonic acid
MPO	myeloperoxidase
MDA	malondialdehyde
PARP	poly ADP-ribose polymerase
NF	nuclear factor
MnSOD	manganese superoxide dismutase
IBD	bowel disorder
ROS	reactive oxygen species
RNS	reactive nitrogen species
TNF-α	alpha tumor necrosis factor
OS	oxidative stress
AOAC	association of official analytical chemists
CAR	carrageenan
IL	interleukin
